# Clinical use of the mRNA urinary biomarker SelectMDx test for prostate cancer

**DOI:** 10.1038/s41391-022-00562-1

**Published:** 2022-07-09

**Authors:** Wieke C. H. Visser, Hans de Jong, Sandra Steyaert, Willem J. G. Melchers, Peter F. A. Mulders, Jack A. Schalken

**Affiliations:** 1 Department of Product Development, MDxHealth BV, Nijmegen, The Netherlands; 2 Department of Computational Biology, Statistics and AI, VOF dobbio, Zelzate, Belgium; 3 Department of Medical Microbiology, Radboud University Medical Center, Nijmegen, The Netherlands; 4 Department of Urology, Radboud University Medical Centre, Nijmegen, The Netherlands

**Keywords:** Prostate cancer, Diagnostic markers

## Abstract

**Background:**

Molecular biomarker tests are developed as diagnostic tools for prostate cancer (PCa) diagnosis. The SelectMDx (MDxHealth, Nijmegen, The Netherlands) test is a urinary-based biomarker test intended to be used to predict presence of high-grade PCa upon biopsy in men with elevated serum prostate-specific antigen (PSA) levels. Previous validation of the SelectMDx test revealed that 53% of the unnecessary biopsies (biopsies indicating no- or GG1 PCa) could be avoided using the SelectMDx test as a decision-tool to select men for prostate biopsy. The objective of this study is to examine the use of the commercially available SelectMDx test under routine, real-life practice.

**Methods:**

Men that underwent a SelectMDx test between May 2019 and December 2020 and that were originating from countries that perform the SelectMDx test on a regular basis were included in this study, resulting in 5157 cases from 10 European countries. Clinical parameters, urinary RNA scores, and test outcomes were compared between PSA groups, age groups, countries, and the validation cohort (described previously [4]) using the Mann–Whitney *U* test, Chi-Square test, Benjamini–Hochberg and Kruskal–Wallis tests.

**Results:**

40.72% of the cases received a negative SelectMDx result. The test is also used in patients outside the intended-use population (PSA < 3 and >10 ng/mL). Clinical parameters (age, PSA density, DRE outcome) varied between patient population from individual countries and the validation cohort, resulting in differences in the potential number of saved biopsies using the test.

**Conclusions:**

The potential number of reduced biopsies in clinical use was 40,72% using the SelectMDx test, assuming a negative SelectMDx test resulted in the decision not to biopsy the patient. This is higher compared to the validation cohort, which is explained by differences in patient population.

## Introduction

The PSA test is widely used as a risk marker for PCa. Increased use of the PSA test resulted in increasing controversy for its use, since the poor specificity of the PSA test results in overdiagnosis and overtreatment of low-risk cancers [1, 2]. The last decade, various molecular biomarker tests have been developed as diagnostic tools for early and non-invasive detection of PCa [3]. The SelectMDx test (MDxHealth, Nijmegen, The Netherlands) is one of these commercially available molecular tests, which predicts presence of high-grade PCa (Gleason score ≥ 7 (GS7)) upon biopsy. The used prediction model includes a molecular risk score based on post-DRE urinary-derived mRNA levels of the *homeobox C6-gene* (HOXC6) and *Distal-Less Homeobox 1*-*gene* (DLX1) combined with clinical variables digital rectal exam (DRE) result, age and PSA density. The test is intended to be used in patients with an abnormal PSA level to help in patient stratification for biopsy, thereby avoiding biopsies [4]. Moreover, using the SelectMDx test to select patients for multiparametric MRI (mp-MRI) is another good strategy, especially in situations with limited accessibility to mp-MRI [5]. Haese et al. [4] validated the SelectMDx test in a multi-center cohort of men waiting for initial biopsy. Of all patients, 35% had a negative SelectMDx result of whom in 95% of the biopsies showed no PCa or PCa GG1. In the subgroup including patients with PSA levels <10 ng/mL, 44% of all cases showed a negative SelectMDx result. Of these patients, again, 95% of the biopsies showed no PCa or PCa GG1. Therefore, 53% of the unnecessary biopsies (biopsies indicating no- or GG1 PCa) could have been avoided when using the SelectMDx test as a decision-tool for performing a biopsy. Since the SelectMDx test is commercially available for a few years now, data is available on the patient group requesting the SelectMDx test. The aim of this study is to obtain insight into the clinical use of the SelectMDx test in daily practice based on 5157 samples from ten European countries.

## Patients and methods

### Urine samples

All data used in this paper is derived from MDxHealth company database. Urine samples of patients were collected between May 2019 to December 2020 under routine practice conditions in several European countries. Urine sample collection and processing was conducted following standardized procedures for the SelectMDx test [6]. Briefly, first-void urine is collected by urologists after DRE (at least three strokes per lobe) and mixed with preservative. After transport (on ambient temperature) to the laboratory in Nijmegen, The Netherlands, urine samples are stored at −20 °C prior to analysis. Next, urinary mRNA expression levels of HOXC6 and DLX1 were quantified using RT-qPCR, followed by normalization for the prostate-specific gene KLK3, resulting in the ‘RNA scores’. Afterward, the SelectMDx RNA scores were combined with clinical variables age, PSA density and DRE result in an algorithm-based prediction model to obtain a patient’s individualized risk on PCa ≥ GS7. For cases reporting unknown prostate volume, risk scores were calculated using a different, previously validated prediction model excluding prostate volume as a variable [4]. Cases originating from countries that do not perform the SelectMDx test on a regular basis (less than 100 cases) were excluded from analysis, resulting in a population of 5157 cases from 10 European countries.

### Statistical analysis

Statistical analysis was performed using R Statistical Software (version 4.0.5; R Foundation for Statistical Computing, Vienna, Austria). The Mann–Whitney *U* test and Pearson Chi-Square test were used to compare respectively, continuous variables (age, PSA, PSA density, prostate volume) and categorial variables (proportions negative/positive SelectMDx results and abnormal/normal DRE result) between the clinical use population and the validation cohort described by Haese et al. [4]. Benjamini–Hochberg test post-hoc analysis was performed to calculate adjusted *p* values for multiple comparison of proportions negative/positive SelectMDx results between each individual country and the validation cohort. Continuous variables of individual countries were compared to the validation cohort using the Kruskal–Wallis multiple comparison post hoc test (Siegel and Castellan [7]). All reported *p* values are compared to 5% significance level.

## Results

The clinical use population (*N* = 5157) showed a median age of 65 years (interquartile range (IQR): 60–71)), a median PSA level of 6.60 ng/mL (IQR: 4.90–9.06) and a median PSA density of 0.13 ng/ml^2^ (IQR: 0.09–0.19). Prostate volume was unknown for 94 cases, resulting in exclusion of these cases in analysis on PSA density. Abnormalities were observed during DRE in 17.82% of the patients. In 40.72% of the cases, the SelectMDx result was negative.

### PSA groups

Although the SelectMDx test is intended to be used in patients with PSA levels between 3 and 10 ng/mL, SelectMDx analysis is also requested for patients with PSA levels below 3 and higher than 10 ng/mL. Therefore, SelectMDx results were analyzed per PSA group. 5.33% of the cases had a PSA value < 3 ng/mL, 76.65% of the cases reported a PSA value ≥ 3 and ≤10 ng/mL, 11.23% of the cases had a PSA value > 10 and ≤15 ng/mL and 6.79% showed a PSA value > 15 ng/mL (see Table 1).

**Table 1:** Data on clinical parameters by PSA group.

PSA group	PSA < 3 ng/mL	PSA ≥ 3 and ≤ 10 ng/mL	PSA > 10 and ≤ 15 ng/mL	PSA > 15 ng/mL	All PSA levels
*N* (% of total)	275 (5.33)	3953 (76.65)	579 (11.23)	350 (6.79)	5157 (100)
Age median, years (Q1–Q3)	62 (54–68)	65 (59–70)	67 (62–72)	67 (62–72)	65 (60–71)
PSA median, ng/mL (Q1–Q3)	1.98 (1.22–2.5)	6.1 (4.88–7.66)	12 (11–13.15)	20 (16.94–24.9)	6.60 (4.90–9.06)
PV median, cm^3^ (Q1–Q3)^a^	37.5 (30–49.25)	50 (38–66)	62.35 (47–88.11)	65 (46.5–100)	51 (38–70)
PSA density median, ng/ml^2^ (Q1–Q3)^a^	0.05 (0.03–0.06)	0.12 (0.09–0.17)	0.19 (0.13–0.28)	0.31 (0.21–0.50)	0.13 (0.09 – 0.19)
DRE result: abnormal %, normal %	29.82; 70.18	15.79; 84.21	19.69; 80.31	28.29; 71.71	17.82; 82.18
RNA score median (Q1–Q3)	27 (14–68.50)	37 (20–71.26)	40 (22–79)	45 (26–85.50)	37.46 (20–73)
SelectMDx result: positive, *N* (%); negative, *N* (%);	68 (24.73); 207 (75.27)	2188 (55.35); 1765 (44.65)	467 (80.66); 112 (19.34)	334 (95.43); 16 (4.57)	3057 (59.28); 2100 (40.72)

^a^Prostate volume and therefore PSA density are unknown for 94 patients.

#### PSA 3–10 ng/mL (*n* = 3953)

**Fig. 1:**
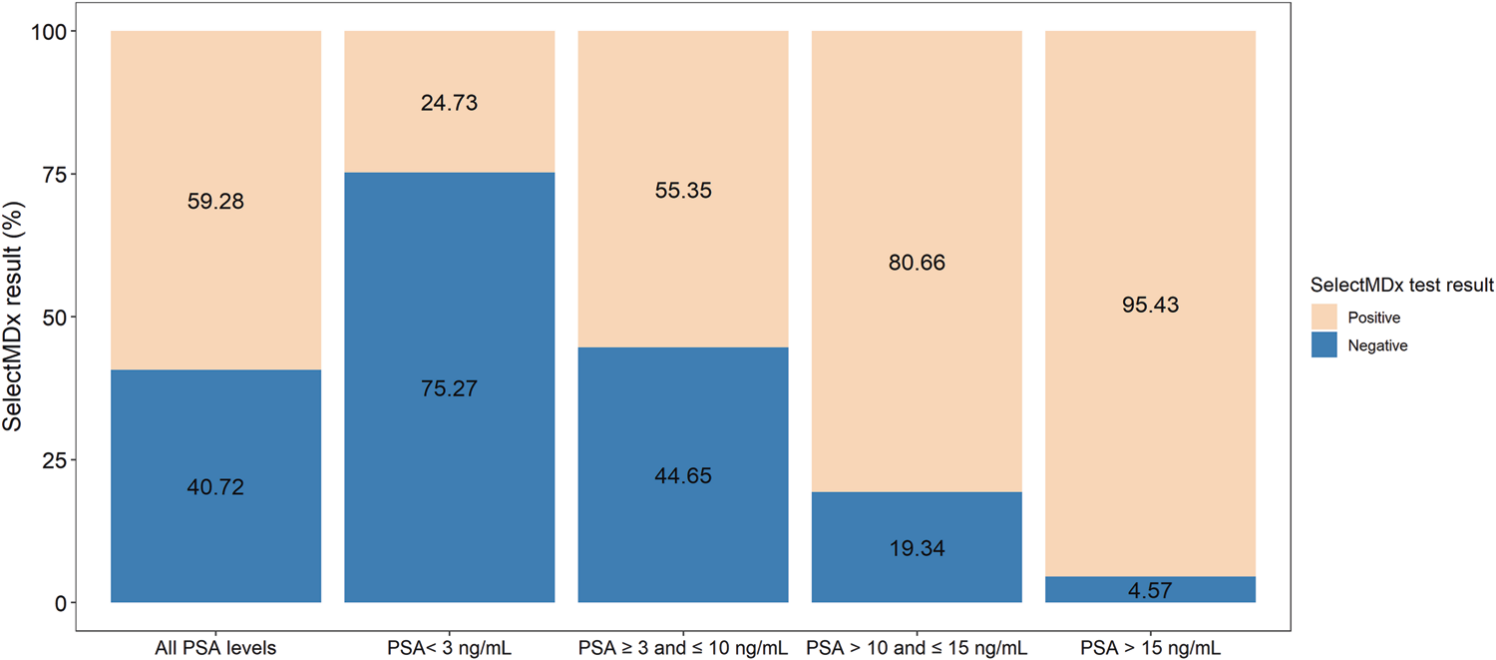
Percentage of SelectMDx negative and positive results by PSA group. PSA = Prostate Specific Antigen; positive = percentage of patients that received a positive SelectMDx test result; negative = percentage of patients that received a negative SelectMDx test result; y = age.

In the intended use population of the SelectMDx test (PSA 3–10 ng/mL), the median age is 65 years (IQR: 59–70), median PSA density is 0.12 ng/ml^2^ (IQR: 0.09–0.17) and 15.79% of the patients showed DRE abnormalities. Analysis of the SelectMDx results per PSA group showed that 44.65% of the cases with PSA between 3–10 received a negative SelectMDx result, therefore biopsies were potentially saved in 1765 men in this intended-use population of the SelectMDx test (see Fig. 1).

#### PSA < 3 ng/mL (*n* = 275)

Age, prostate volume, PSA density and RNA score were lower compared to the other PSA groups. In contrast, the percentage of cases showing an abnormal DRE result is relatively high (29.82%), as compared to the patients with PSA between 3 and 10 (15.79%). A relatively high percentage of patients (75.27%) in this group received a negative SelectMDx result (Fig. 1). In patients with a PSA level below 3 and an abnormal DRE finding, the SelectMDx test reported 35 SelectMDx positive cases (43.67%) and 49 SelectMDx negative cases (58.33%).

#### PSA > 10 ng/mL (*n* = 929)

Age, prostate volume, and RNA score are higher in this group compared to the other PSA groups. Prostate volume slightly increases between the groups of PSA 10–15 and PSA > 15, in contrast to larger differences in PSA between both groups. This results in large differences in PSA density between these groups (0.19 ng/ml^2^ versus 0.31 ng/ml^2^). In cases with PSA levels > 10 and ≤15, only 19.34% (*n* = 112) received a negative result. The lowest percentage of negative results (4.57%) is found in patients with PSA levels > 15 ng/mL (*n* = 16), see Fig. 1.

### Age groups

**Fig. 2:**
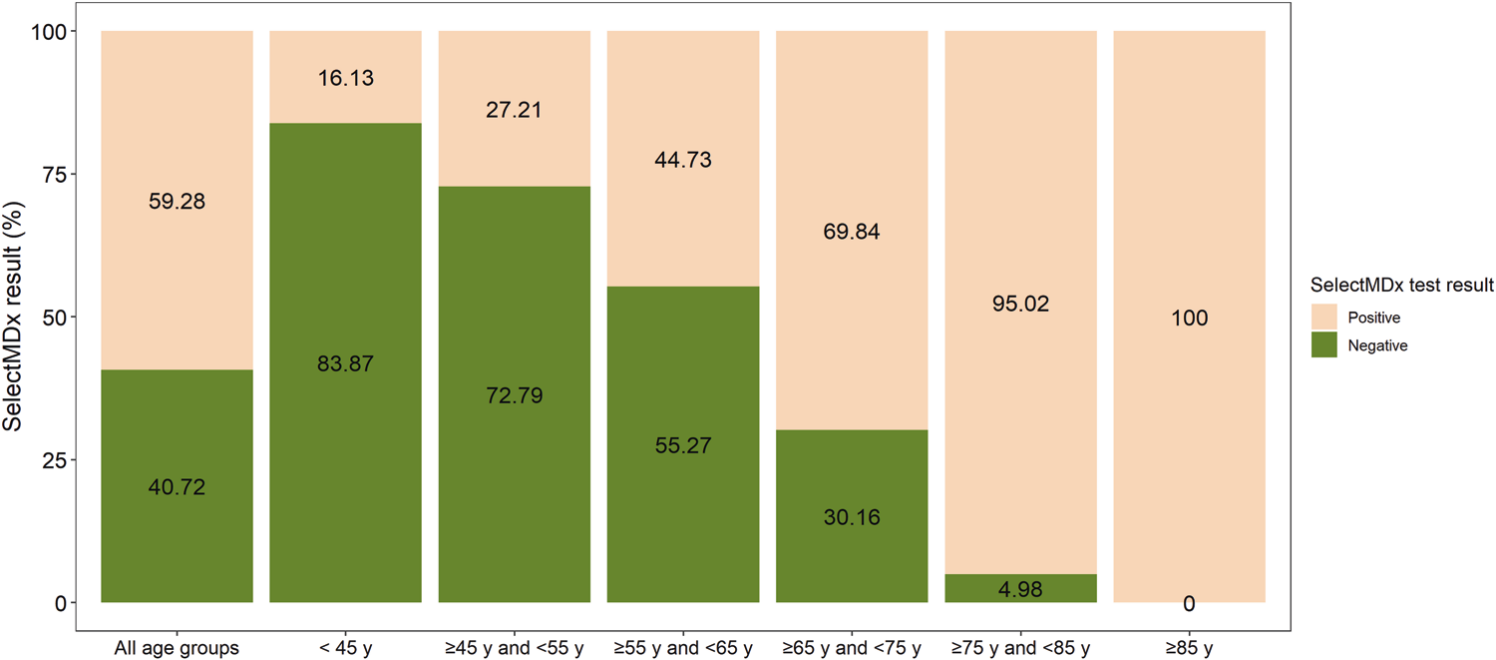
Percentage of SelectMDx negative and positive results by age group. Positive =percentage of patients that received a positive SelectMDx test result; negative = percentage of patients that received a negative SelectMDx test result.

Next, subgroup analysis was performed to compare data on clinical risk factors and SelectMDx results by age groups. Most of the patients have an age ≥65 and <75 (44.23%), followed by the age group ≥55 and <65 (36.77%) (see Table 2). Prostate volume (median), RNA score (median) and percentage abnormal DRE findings increase with increasing age group (except for prostate volume in two highest age groups). PSA density was shown to be stable over the age groups <75 years (PSA density 0.12–0.13 ng/ml^2^) and showed a sharp increase in age group 75–85 years (PSA density 0.15 ng/ml^2^) and age group >85 years (PSA density 0.24 ng/ml^2^). This can be explained by the combination of increasing PSA and a stable prostate volume. Although the PSA density and percentage abnormal DRE findings between age group 45–55 years and 55–65 years are similar, the percentage of cases with a positive SelectMDx result is 17.53% higher in the 55–65 years age group. This could be explained by the increase in age and RNA score in the 55–65 years age group. Overall, the percentage negative SelectMDx results decrease with age, ranging from 83.87% negative results in patients aged <45 years to 0% negative SelectMDx results in patients aged >85 years (see Fig. 2). However, age is a confounding factor in this last analysis since age is included in the SelectMDx algorithm.

**Table 2:** Data on clinical parameters by age group.

Age group	<45 years	≥45 years and <55 years	≥55 years and <65 years	≥65 years and <75 years	≥75 years and <85 years	≥85 years	All age groups
*N* (%)	31 (0.6)	430 (8.34)	1896 (36.77)	2281 (44.23)	502 (9.73)	17 (0.33)	5157 (100)
Age median, years (Q1–Q3)	42 (39.5–44)	52 (50–53)	60 (58–62)	69 (67–71)	77 (76–79)	87 (86–89)	65 (60–71)
PSA median, ng/mL (Q1–Q3)	3.3 (2.05–5.48)	5.33 (3.86–7.30)	6.27 (4.80–8.57)	6.81 (5.10–9.35)	7.93 (5.90–10.83)	9.76 (6.16–14.63)	6.60 (4.90–9.06)
PV median, cm^3^ (Q1–Q3)^a^	25.5 (20.5–30)	40 (30–55.75)	50 (38.7–66)	55 (40–75)	52 (40–72.5)	55 (41.5–81)	51 (38–70)
PSA density median, ng/ml^2^ (Q1–Q3)^a^	0.13 (0.07–0.24)	0.12 (0.09–0.18)	0.13 (0.09–0.19)	0.12 (0.09–0.19)	0.15 (0.1–0.22)	0.24 (0.12–0.75)	0.13 (0.09–0.19)
DRE result: abnormal %, normal %	6.45; 93.55	14.42; 85.58	14.93; 85.07	18.37; 81.63	28.49; 71.51	58.82; 41.18	17.82; 82.18
RNA score median (Q1–Q3)	13 (7.39–29.50)	22 (12–39.77)	31 (17–59)	43 (24–83.73)	62 (35.16–109.75)	135 (80.42–307.00)	37.46 (20–73)
SelectMDx result: positive, *N* (%); negative, *N* (%);	5 (16.13); 26 (83.87)	117 (27.21); 313 (72.79)	848 (44.73); 1048 (55.27)	1593 (69.84); 688 (30.16)	477 (95.02); 25 (4.98)	17 (100); 0 (0)	3057 (59.28); 2100 (40.72)

^a^Prostate volume and therefore PSA density unknown for 94 patients.

### Comparing countries and the validation cohort

Subgroup analysis was performed comparing patient groups from different countries (see Table 3). Most of the requests for SelectMDx tests originated from the Netherlands (34.83%) followed by Spain (31.12%) and Italy (7.27%). Differences were found in patient characteristics between countries, with PSA (median) ranging from 5.87 ng/mL in Slovenia to 7.67 ng/mL in Belgium and median age ranging from 63 years in Belgium to 69 years in France. PSA density varied from 0.11 ng/ml^2^ (Italy) to 0.16 ng/ml^2^ (Belgium). Considering DRE outcome, cases from Switzerland (45.3%) and Germany (36.9%) have higher percentage abnormal DRE, compared to all other countries (17.82%). PSA density ranges from 0.11 ng/ml^2^ (Italy) to 0.16 ng/ml^2^ (Belgium). Next, the percentage of cases with PSA levels below 3 ng/mL varies from 2.43% in Spain to 12.79% in Poland. These differences in patient characteristics between countries are translated into differences in test results: the percentage negative SelectMDx test results range from 30.3% in France to 46.93% in Italy.

**Table 3:** Data on clinical parameters by country.

Country	Netherlands	Spain	Italy	Germany	Romania	Slovenia	Poland	Belgium	France	Switzerland
*N*	1796	1605	375	312	221	220	219	171	132	106
Age, median (Q1–Q3)	66 (61–71)	65 (59–70)	65 (59–70)	65 (60–71.25)	64 (58–69)	66 (59–71)	64 (57–68)	63 (58–68.5)	69 (63.75–73)	65 (58–70.75)
PSA, median (Q1–Q3)	6.66 (4.9–8.7)	6.69 (5.1–9.3)	6.4 (4.7–9.1)	6.68 (4.8–10.01)	5.93 (4.3–8.87)	5.87 (4.45–8.2)	6 (4.41–8.95)	7.67 (5.76–10.65)	6.73 (4.5–9.45)	6.75 (4.9–8.91)
PV, median (Q1–Q3)^a^	51 (40–70)	51 (40–71)	60 (44.96–77.25)	47 (35–68.6)	50 (35–68.25)	44.5 (34–63.5)	45 (33–60.5)	49 (36.25–65)	50 (40–70)	53 (40–65)
DRE abnormal (%)	11.86	13.96	26.4	36.86	31.67	15	32.42	11.7	19.7	45.28
PSA density median (Q1–Q3)^a^	0.12 (0.09–0.18)	0.13 (0.1–0.2)	0.11 (0.07–0.16)	0.14 (0.09–0.22)	0.12 (0.08–0.19)	0.13 (0.09–0.18)	0.13 (0.08–0.21)	0.16 (0.11–0.22)	0.14 (0.09–0.22)	0.12 (0.09–0.18)
Cases PSA < 3 (%)	5.12	2.43	10.4	8.65	8.14	6.36	12.79	2.92	9.85	5.66
Cases PSA 3–5 (%)	22.16	21.06	21.33	19.23	26.7	29.09	20.09	15.2	19.7	19.81
Cases PSA 5–10 (%)	58.57	56.2	50.13	47.12	48.42	50.91	49.32	53.22	48.48	56.6
Cases PSA 10–15 (%)	8.41	13.02	11.2	14.74	10.41	10.91	12.33	16.96	14.39	8.49
Cases PSA > 15 (%)	5.73	7.29	6.93	10.26	6.33	2.73	5.48	11.7	7.58	9.43
RNA score	38 (21–74.78)	37 (20–69)	35 (18.5–70.06)	39.46 (22–81.25)	47 (20–94)	34 (18–71)	33.04 (19.99–68.41)	33.97 (19–65.5)	43 (24–79.56)	27 (14.01–68.75)
SelectMDx result: positive, *N* (%); negative, *N* (%);	1 070 (59.58); 726 (40.42)	929 (57.88); 676 (42.12)	199 (53.07); 176 (46.93)	212 (67.95); 100 (32.05)	137 (61.99); 84 (38.01)	126 (57.27); 94 (42.73)	125 (57.08); 94 (42.92)	101 (59.06); 70 (40.94)	92 (69.7); 40 (30.3)	66 (62.26); 40 (37.74)

^a^Prostate volume and therefore PSA density unknown for 94 patients.

In the validation cohort (all PSA levels) of the SelectMDx test, 35.70% of all SelectMDx tests resulted in a negative result. When comparing the ratio of positive/negative SelectMDx result between the clinical use population and the validation cohort, the number of cases with a negative SelectMDx result was significantly higher in the clinical use population compared to the validation cohort: χ2 (1) = 8.18, *p* < 0.05 (see Table 4). No significant differences were found in PSA levels and age between the clinical use population and the validation cohort. In contrast, prostate volume was significantly higher in the clinical use population compared to the validation cohort, and therefore, PSA density was significantly lower in clinical use cohort compared to the validation cohort. The validation cohort included significantly more cases with an abnormal DRE result as compared to the clinical use population.

**Table 4:** Clinical use population compared to validation cohort.

	Clinical use population	Validation cohort Haese [4]	*P* value
*N*	5 157	916	
Age median, years (Q1–Q3)	65 (60–71)	65 (60–70)	0.465, ^a^
PSA median, ng/mL (Q1–Q3)	6.6 (4.90–9.06)	6.37 (4.50–9.20)	0.067, ^a^
PV median, cm^3^ (Q1–Q3)^b^	50 (38–70)	42.5 (30.9–60.0)	<0.001*, ^a,b^
PSA density median, ng/ml^2^ (Q1–Q3)	0.126 (0.09–0.19)	0.140 (0.09–0.22)	<0.001*, ^a,b^
DRE abnormal/normal/unknown (%)	919 (17.82)/4 238 (82.18)/0 (0)	204 (22.28)/706 (77.07)/6 (0.66)	0.001*, ^c,d^
PSA < 3 ng/ml, *N* (%)	275 (5.33)	80 (8.73)	<0.001*, ^c^
PSA ≥ 3 & ≤10 ng/ml, *N* (%)	3 953 (76.65)	640 (69.87)	<0.001*, ^c^
PSA above 10 ng/ml, *N* (%)	929 (18.01)	196 (21.40)	0.015*, ^c^
SelectMDx result: positive, *N* (%); negative, *N* (%);	3 057 (59.28)/2 100 (40.72)	589 (64.30)/327 (35.70)	0.004*, ^c^

*Significant difference (*p* < 0.05) between both groups.

^a^Mann–Whitney *U* Test.

^b^Prostate volume and therefore PSA density unknown for 94 patients in the clinical use population and for 9 patients in the validation cohort Haese [4].

^c^Pearson Chi-Square Test.

^d^6 cases from validation cohort excluded, because DRE outcome was unknown.

**Fig. 3:**
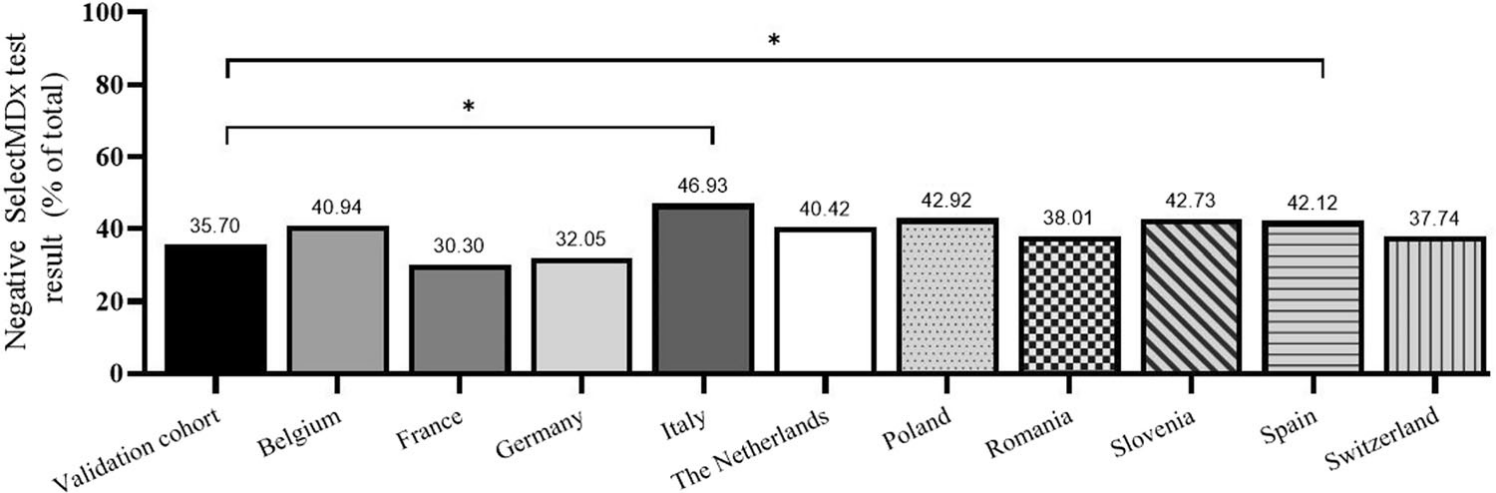
Percentage of SelectMDx negative results by country and the validation cohort. ^∗^Adjusted *p* value < 0.05.

Next, the ratio positive and negative SelectMDx test results were compared between each country and the validation cohort. A significantly higher percentage negative SelectMDx result was observed in Italy (χ2 (1) = 14.12, adjusted *p* < 0.05) and Spain (χ2 (1) = 10.03, adjusted *p* < 0.05) compared to the validation cohort (Fig. 3). These differences in SelectMDx results could be partly explained by differences in patients selected for a SelectMDx test in these countries. Post-hoc analysis on Italy and Spain showed that PSA density is significantly lower in Spain and Italy compared to the validation cohort. However, PSA is significantly higher in Spain compared to the validation cohort. PSA density is the lowest in Italy, contributing to the higher number of negative SelectMDx results in Italy (see Supplementary Data).

## Discussion

During the development and optimization of biomarker tests, studies are conducted including a defined patient population, resulting in test performance characteristics for a specified population. For SelectMDx test, validation studies included men waiting for biopsy having an elevated PSA [4, 8]. The SelectMDx test is intended to be used after a PSA test in patients with a PSA level between 3 and 10 ng/mL and who did not undergo a biopsy yet to guide clinicians in further (biopsy) decision-making. This paper provides a detailed insight into the clinical use of the SelectMDx test in routine practice. Data on SelectMDx test results, clinical risk factors and RNA scores are compared in more detail between PSA groups, age groups and countries.

Performing the SelectMDx test for all patients resulted in a positive SelectMDx result for 59.28% of the patients and a corresponding 40.72% of the patients received a negative result. Therefore, the potential number of reduced biopsies is 40.72% using the SelectMDx test, considering a negative SelectMDx test resulted in the decision not to biopsy the patient. Another clinical utility study on biomarker tests for PCa showed similar results for the urinary ExosomeDx Prostate IntelliScore (potentially saving 39.9% of unnecessary biopsies) and less saved biopsies with the 4K-score (29.5%) [9]. In our study, most cases (>75%) had a PSA between 3 and 10 ng/mL. Using the SelectMDx test as a decision tool for performing a subsequent biopsy and a negative test result will lead to the decision not to perform a biopsy, most biopsies can be saved in patients with PSA below 10 ng/mL.

Although it is not common to take further diagnostic actions in men with PSA below 3 ng/mL, 275 SelectMDx requests were obtained from patients in this PSA group. Multiple factors might play a role in the clinicians’ decision to request a SelectMDx test for these patients, e.g., patients request for further diagnostic actions, urologist is not well informed on the recommended intended population of the test or aberrant clinical parameters other than PSA value were observed, such as a family history of PCa or abnormalities observed during DRE. The last factor is observed in current data: the percentage of patients with an abnormal DRE result is relatively high in the patient group with PSA < 3 ng/mL (29.28%) as compared to the patient group with PSA levels between 3 and 10 ng/mL (15.79%). Using SelectMDx in this subgroup (PSA < 3 and abnormal DRE) showed that 58.33% could have been saved. On the other hand, performing the SelectMDx test for patients with PSA levels higher than 15 ng/mL was shown to be less useful, since almost all these requests (>95%) resulted in a positive result.

Shore and co-authors examined the clinical utility of the SelectMDx test as a decision tool for performing biopsy in men waiting for initial biopsy in the United States [6]. Of all patients (*n* = 418) with a negative SelectMDx result, 12.5% had a subsequent biopsy, in contrast to 60.7% of the patients with a positive SelectMDx result, indicating that the SelectMDx results affects biopsy decision making procedures. In addition, time between SelectMDx test and biopsy was significantly shorter for SelectMDx positive cases compared to SelectMDx negative cases. A larger percentage of these men showed a negative SelectMDx test (61%) compared to the European clinical use population evaluated in this paper (40.72%) and the validation cohort (35.70%) which might be explained by a higher median PSA level in the European clinical use population (PSA: 6.6 ng/mL) and the validation cohort (PSA: 6.37 ng/mL) compared to the US clinical utility population (PSA: 5.1 ng/mL).

Current analysis comparing SelectMDx test results and clinical risk factor values (PSA, PSA density, age, and abnormal DRE) by country, showed differences on all these risk factors and SelectMDx outcome. This could indicate that the diagnostic path for PCa diagnosis varies between countries, which would result in differences in patient populations requesting SelectMDx tests. Complementary use of the SelectMDx test with other diagnostic tools for early detection of PCa, such as other blood- or urinary biomarker tests, risk calculators or mp-MRI is likely. The EAU guidelines recommend the use of risk calculators or imaging tools in risk-assessment of men with elevated PSA levels (2–10 ng/mL) and a normal DRE result [10]. Variation in diagnostic tools used between or within countries could affect the patient population for SelectMDx in routine practice and therefore the number of biopsies saved by the SelectMDx test. The complementary use of the SelectMDx test with other tools was studied before. Maggi et al. [11] examined several diagnostic strategies incorporating SelectMDx test and mp-MRI for detecting PCa and clinically significant PCa. Combining mp-MRI with the SelectMDx showed best performance for detecting PCa and cs-PCa, when compared to the combinations of MRI and either PSA or PSA density. The authors concluded as best strategy that biopsies should be taken if initial MRI is positive (PI-RADS 4–5) or if SelectMDx test is positive, which should be conducted after negative MRI [11]. The potential of combining SelectMDx and mp-MRI was also demonstrated by Hendriks et al. [5], although these authors opted to use SelectMDx first, prior to mp-MRI to avoid biopsies.

A limitation of this study is the missing information on biopsy decision following a SelectMDx test result. To obtain insight in the effect of a (negative) SelectMDx outcome on further decision-making regarding biopsy, and in fact biopsy-outcome, more information is required. Therefore, the clinical utility as such cannot be assessed and was not considered a primary aim of this study.

## Conclusion

To conclude, between May 2019 and December 2020, 5175 urine samples were analyzed by the SelectMDx test and tested in this study. The potential number of reduced biopsies was 40.72% using the SelectMDx test, assuming a negative SelectMDx test resulted in the decision not to biopsy the patient. This number is higher compared to the cohort in which the SelectMDx test was validated, which can be explained by the significant lower numbers of cases with abnormal DRE and lower PSA density in the clinical use population. Although the intended-use population for the SelectMDx test is patients with a PSA level between 3 and 10 ng/mL, SelectMDx tests were requested for patients with other PSA levels. Using the SelectMDx as decision tool in patients with a PSA < 3 ng/mL, the number of potentially saved biopsies increased to 75.27%. Next, variation in patient groups were observed between countries, which affects the ratio positive/negative SelectMDx results and therefore the number of biopsies saved using the SelectMDx test.

## Supplementary information

Supplementary data

## Data Availability

The datasets cannot be made openly available due to its proprietary nature. An extended description of the data from validation cohort can be found at 10.1097/JU.0000000000000293.
