# Supplementary figures and images for: Clinical use of the mRNA urinary biomarker SelectMDx test for prostate cancer
Source: Prostate Cancer Prostatic Dis. 2022 Jul 9;25(3):583–9. doi: 10.1038/s41391-022-00562-1 (PMC9385481; doi:10.1038/s41391-022-00562-1)

Supplementary data: 1


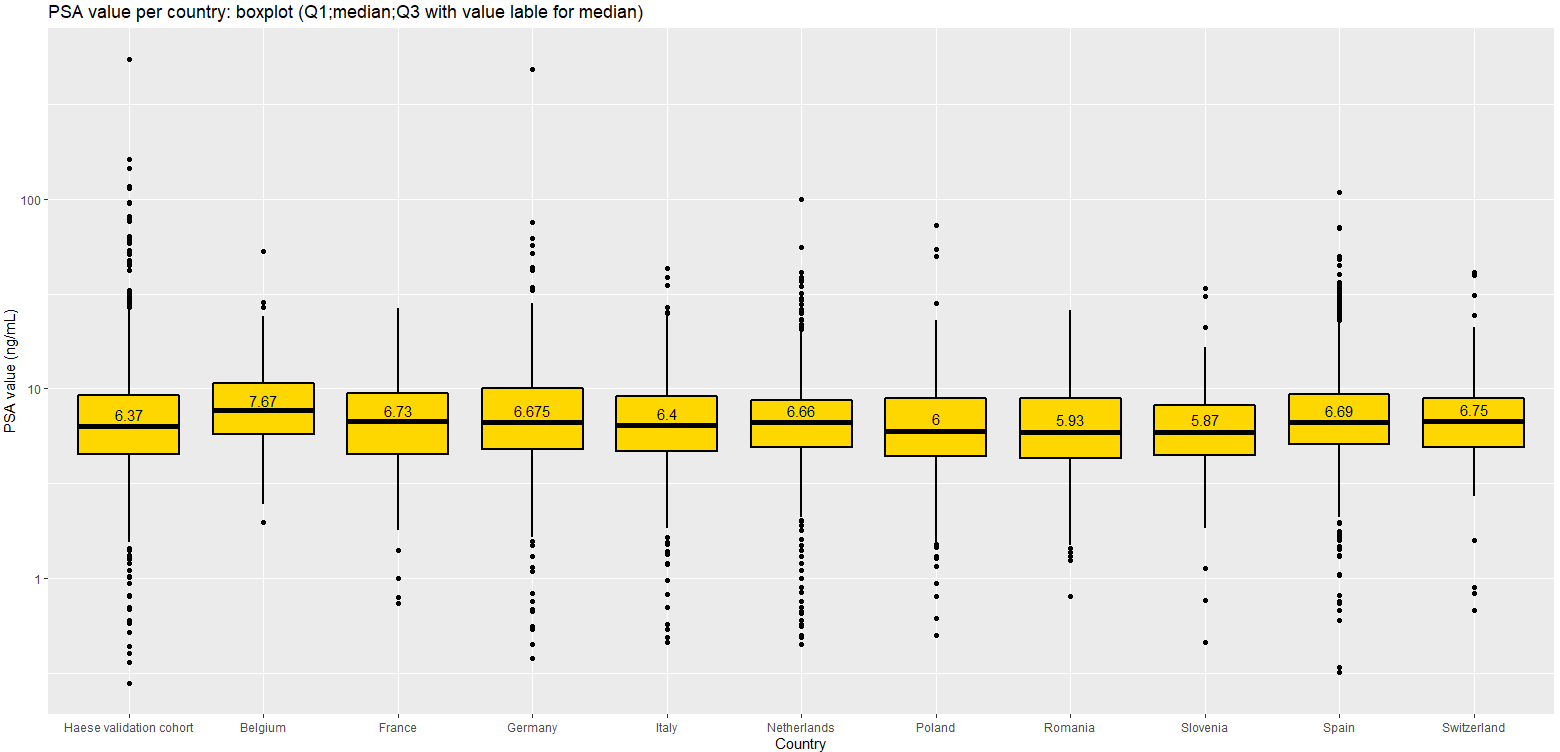

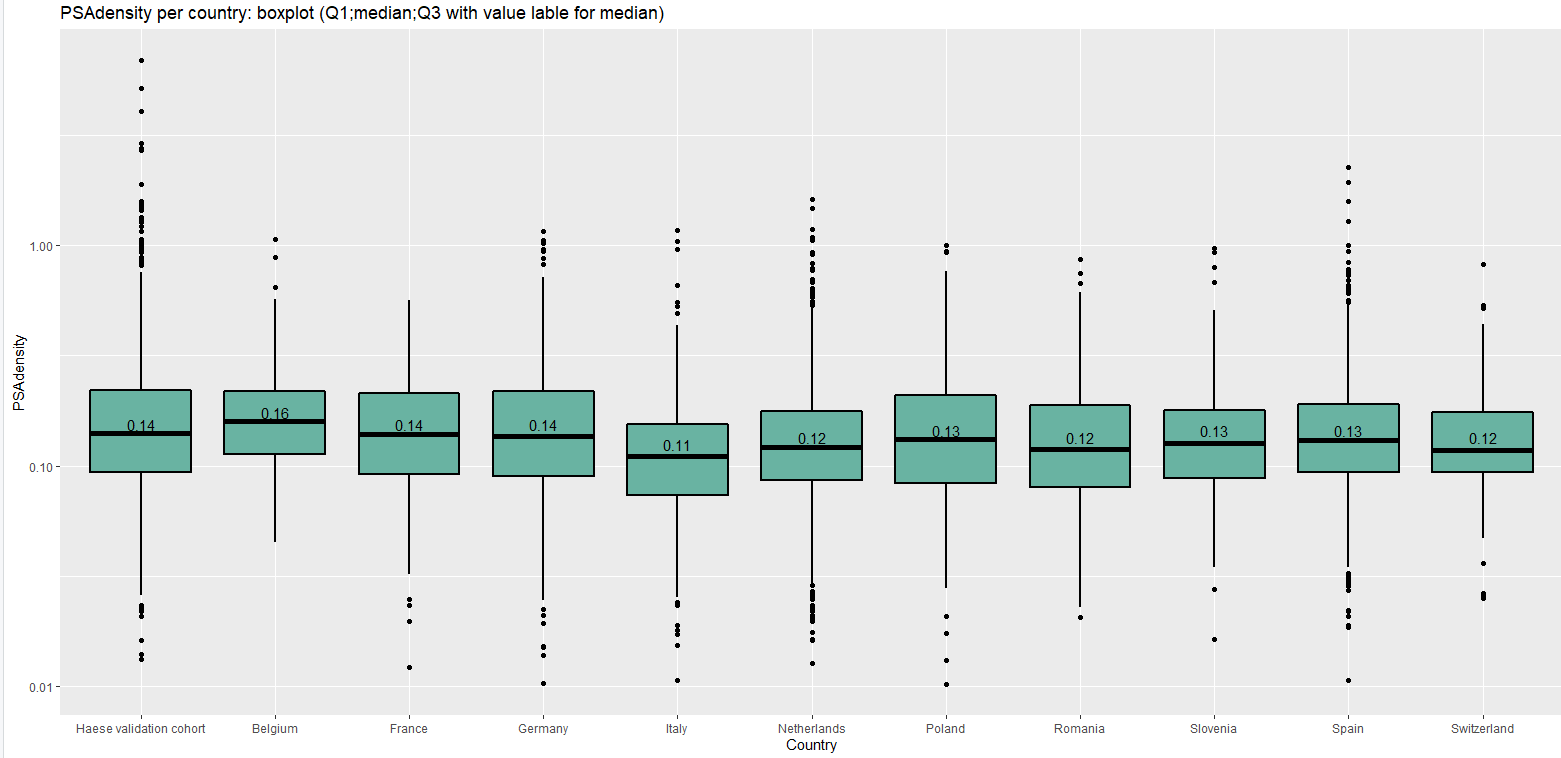


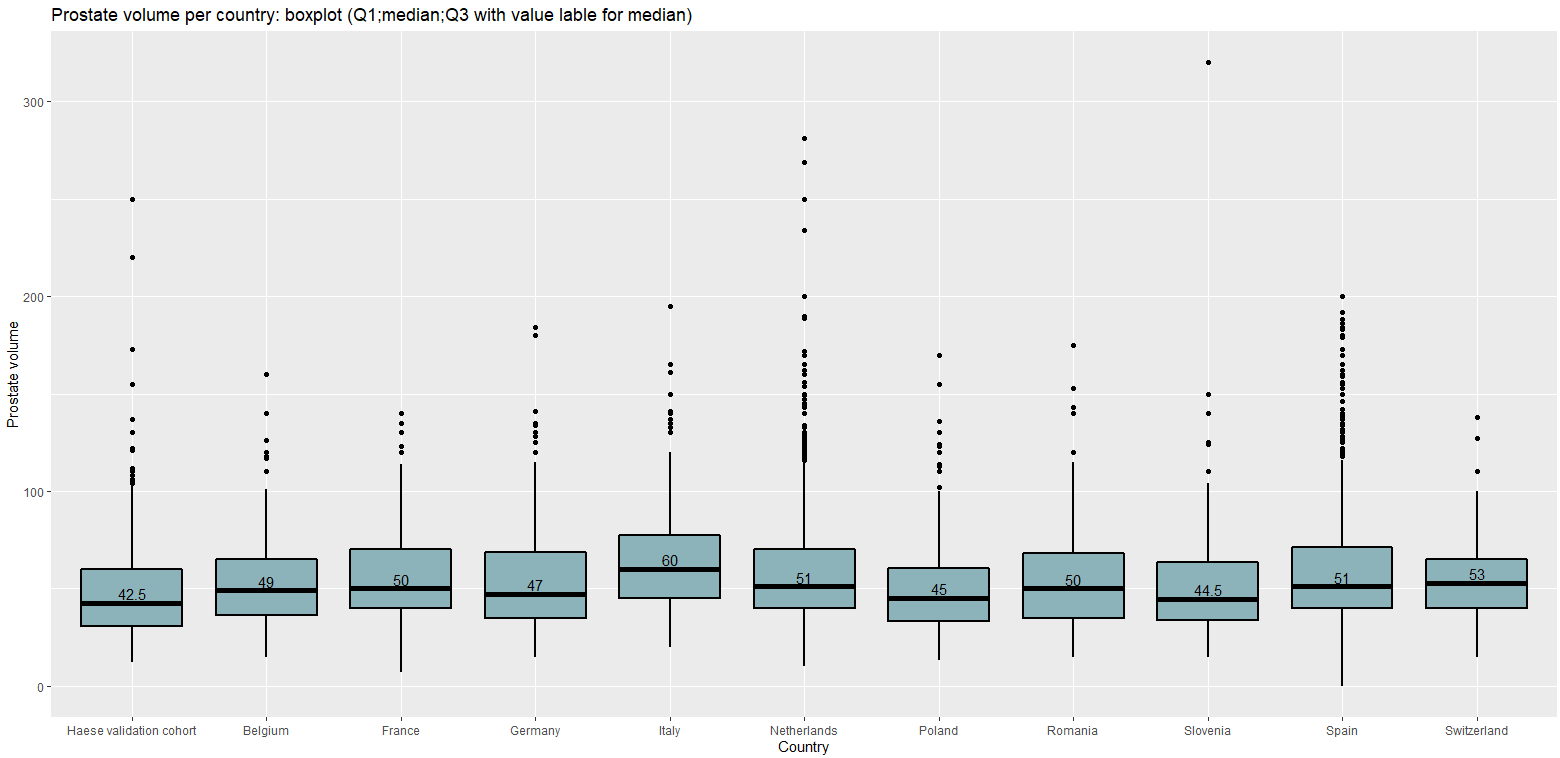

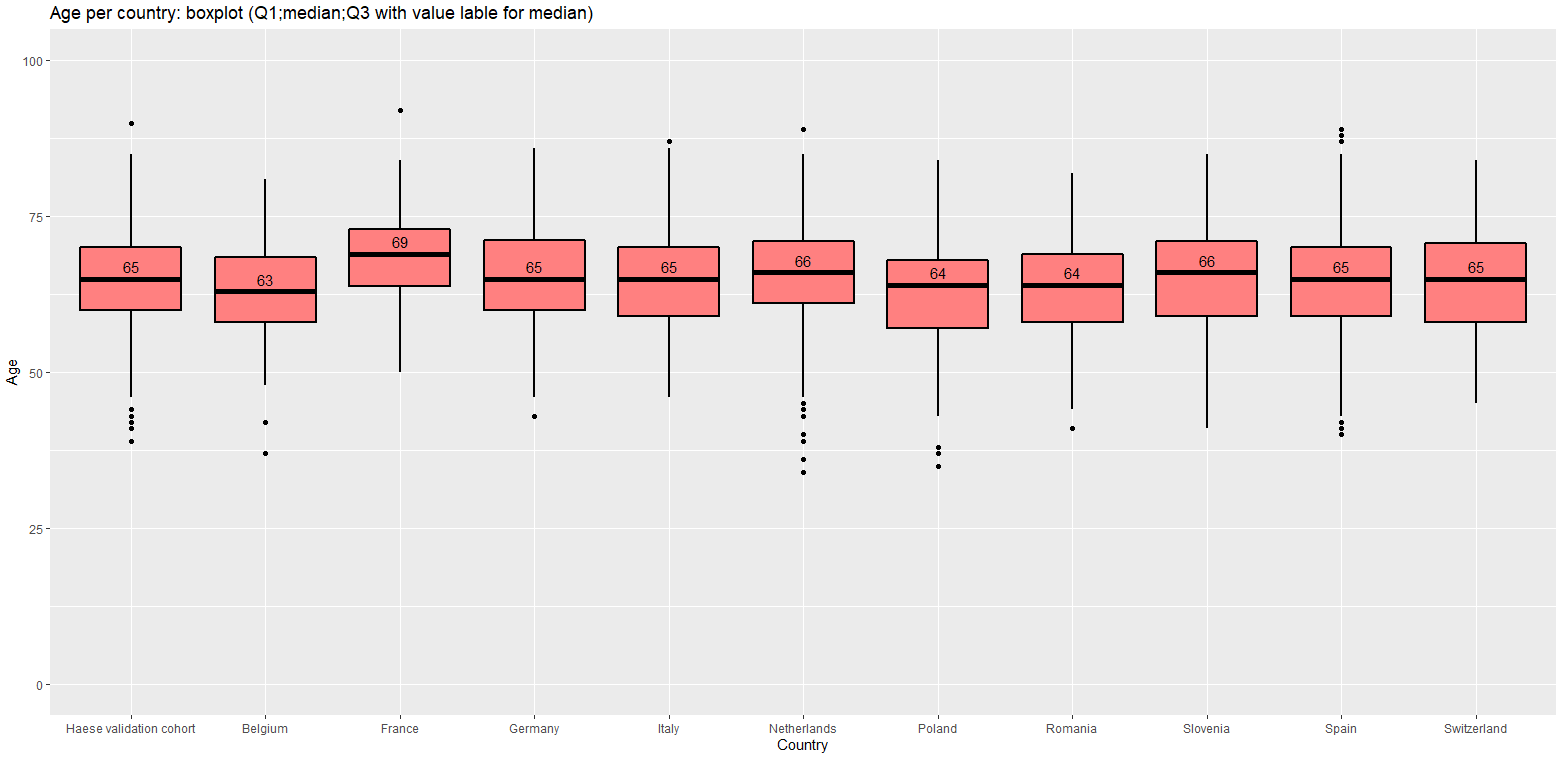

Supplement: Supplementary file 1 — Supplementary data [file 41391_2022_562_MOESM1_ESM.docx]
